# 184. Impact of the BioFire® FilmArray® Blood Culture Identification 2 Panel on Antimicrobial Treatment of Enterobacterales Blood Stream Infections

**DOI:** 10.1093/ofid/ofac492.262

**Published:** 2022-12-15

**Authors:** Ashley Logan, James Beardsley, Alex D Taylor, John C Williamson, Elizabeth Palavecino, Vera Luther, Christopher Ohl, Tyler Stone

**Affiliations:** University of Kentucky HealthCare, Lexington, Kentucky; Atrium Health Wake Forest Baptist, Winton-Salem, North Carolina; Atrium Health Wake Forest Baptist, Winton-Salem, North Carolina; Atrium Health Wake Forest Baptist, Winton-Salem, North Carolina; Wake Forest School of Medicine, Winston Salem, North Carolina; Wake Forest School of Medicine, Winston Salem, North Carolina; Wake Forest School of Medicine, Winston Salem, North Carolina; Atrium Health Wake Forest Baptist, Winton-Salem, North Carolina

## Abstract

**Background:**

The BioFire® FilmArray® Blood Culture Identification 2 (BCID2) panel is a rapid diagnostic tool that uses multiplex polymerase chain reaction technology to identify 43 different genetic targets. BCID2 detects 13 additional targets, including 7 resistance markers, not detected by the original BCID panel. For this reason, our institution changed panels from BCID to BCID2 in August 2020. The purpose of this study was to assess the time to antibiotic change for Enterobacterales bloodstream infections before and after switch to BCID2.

**Methods:**

This was a retrospective, observational, before-and-after study conducted at an 885-bed academic medical center. Adult patients with blood cultures positive for *E. coli* or *K. pneumoniae* (EC/KP) who received broad spectrum beta-lactam therapy were included. Patients with a polymicrobial or concurrent infection, hemodynamic instability, lack of source control by day 5, or death within 72 hours (hrs) of cultures being collected were excluded. The primary outcome was time to antibiotic change in the BCID group compared to the BCID2 group. Secondary outcomes included time to de-escalation or escalation and the number of opportunities for de-escalation or escalation.

**Results:**

88 patients were included, 55 in the BCID-group and 33 in the BCID2-group. Baseline characteristics are reported in Table 1. Median time to first antibiotic change was 65.3 hrs in the BCID-group vs 69.6 hrs in the BCID2-group (p=0.53). Times to first de-escalation and escalation and number of opportunities for de-escalation and escalation are presented in Table 2. Proportion of missed de-escalation opportunities based on panel results was greater in the BCID2 group (63.6% vs 0%, p< 0.05).

**Figure:**
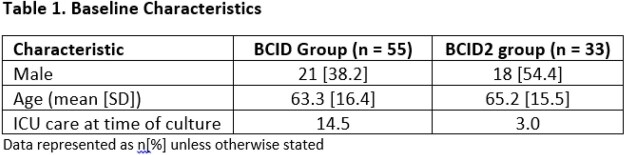

**Figure:**
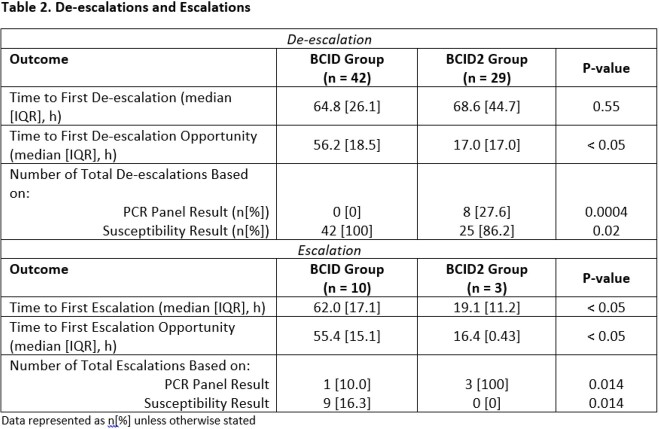

**Conclusion:**

Opportunities to make timely antibiotic modifications with the use of the BCID2 panel exist for EC/KP blood stream infections. While no difference in time to first antibiotic change was present between groups, this highlights the importance of having an active stewardship team member available to respond to rapid diagnostic test results.

**Disclosures:**

**All Authors**: No reported disclosures.

